# Factors impacting on the activation and approach times of helicopter emergency medical services in four Alpine countries

**DOI:** 10.1186/1757-7241-20-56

**Published:** 2012-08-20

**Authors:** Iztok Tomazin, Miljana Vegnuti, John Ellerton, Oliver Reisten, Guenther Sumann, Janko Kersnik

**Affiliations:** 1 Mountain Rescue Association of Slovenia, Bleiweissova 34, 4000, Kranj, Slovenia; 2 HEMS Slovenia, Gosposvetska 9, 4000, Kranj, Slovenia; 3 International Commission for Mountain Emergency Medicine (ICAR Medcom), Zermatt, Switzerland; 4 University Clinic of Respiratory and Allergic Diseases Golnik, Golnik 36, 4204, Golnik, Slovenia; 5 Medical Officer, Mountain Rescue (England & Wales), 1, Leith Close, Cliburn, Penrith, Cumbria, CA10 3AJ, England; 6 Air Zermatt Air and Mountain Rescue, Alpine Rescue Center, CH-3920, Zermatt, Switzerland; 7 HEMS Christophorus 4, Kitzbuehel, Austria; 8 Department of family medicine, Medical School, University of Ljubljana, Poljanski nasip 58, 1000, Ljubljana, Slovenia; 9 Department of family medicine, Medical School, University of Maribor, Slomrtme trg 15, 2000, Maribor, Slovenia

**Keywords:** Emergency medical services, Air ambulances, Emergency helicopters, Quality of health care, Activation and approach time

## Abstract

**Background:**

The outcome of severely injured or ill patients can be time dependent. Short activation and approach times for emergency medical service (EMS) units are widely recognized to be important quality indicators. The use of a helicopter emergency medical service (HEMS) can significantly shorten rescue missions especially in mountainous areas. We aimed to analyze the HEMS characteristics that influence the activation and approach times.

**Methods:**

In a multi-centre retrospective study, we analyzed 6121 rescue missions from nine HEMS bases situated in mountainous regions of four European countries.

**Results:**

We found large differences in mean activation and approach times among HEMS bases. The shortest mean activation time was 2.9 minutes; the longest 17.0 minutes. The shortest mean approach time was 10.4 minutes; the longest 45.0 minutes. Short times are linked (p < 0.001) to the following conditions: helicopter operator is not state owned; HEMS is integrated in EMS; all crew members are at the same location; doctors come from state or private health institutions; organization performing HEMS is privately owned; helicopters are only for HEMS; operation area is around 10.000 km2; HEMS activation is by a dispatching centre of regional government who is in charge of making decisions; there is only one intermediator in the emergency call; helicopter is equipped with hoist or fixed line; HEMS has more than one base with helicopters, and one team per base; closest neighboring base is 90 km away; HEMS is about 20 years old and has more than 650 missions per year; and modern helicopters are used.

**Conclusions:**

An improvement in HEMS activation and approach times is possible. We found 17 factors associated with shorter times.

## Background

Following a medical emergency, the time from incident to pre-hospital care being started, and time to definitive hospital medical care are considered to be influential factors determining patient outcome [1-3]. This is particularly so for a severely injured or ill patient, where delay can compromise recovery or survival [4,5]. Emergencies in remote and mountainous areas have an inherently longer pre-hospital phase compared with urban emergencies. This raises the possibility that a substantial improvement in outcome may be achieved by rapid on-site medical treatment and transport to the nearest appropriate medical facility utilizing a helicopter [6-15]. Modern emergency medical services (EMS) incorporating a helicopter (HEMS) attempt to do this by striving to achieve a short activation (from emergency call to HEMS team take-off) and approach time (from emergency call to arriving at the emergency site) [16]. These times indicates the system’ysability to respond to an emergency in a timely manner and are recognized to be among the most important and easily measured quality indicators in pre-hospital emergency medicine [2,17-20]. An efficient HEMS system is combination of expensive advanced technology (helicopters, communication etc.) and, even more importantly, a well organized, trained and educated human resource (helicopter crew, dispatch centers crew etc.) [16,21,22].

An activation time of 5 or less minutes and an approach time of less than 20 minutes has been suggested as ideal goal even in mountainous areas. A minimal standard should be 'as fast as feasible without compromising safety' [1,16,22]. But as HEMS missions in the mountains are especially challenging and place unique demands on the persons, organizations and resources involved [7], safety must be the highest priority in mountain rescue [16].

The aim of this study was to measure current activation and approach times in participating HEMS bases operating in some mountain areas of Europe, to compare them and to investigate factors associated with any variations in these times. This information has not been published previously and could be helpful to HEMS organizations in appraising their performance.

## Methods

We conducted a retrospective observational study of 6121 consecutive missions from nine HEMS bases from four countries (Slovenia, Austria, Switzerland and Spain) during the years 2006–2009. The study was limited to daytime and primary missions (as secondary and night-missions have differing time constraints). Search missions and rescues of uninjured or healthy people were excluded.

Data on 32 variables (factors) thought to have an impact on activation and approach times was collected by official reports of the work of the bases and by two questionnaires designed specifically for the study. Participating bases were chosen by invitation following a request to members of the International Commission for mountain emergency medicine (ICAR Medcom) and are listed in Table 1. All participating bases had to operate in a mountainous area though the proportion of rescue missions performed in mountainous areas, as opposed to flat or urban terrain, was not defined.

**Table 1:** Main features of participating HEMS bases

**Base**	**Country**	***Helicopter operator***	***Working area km***^***2***^	***HEMS only***	***Missions included***	**Average missions/yr**	***No. of mediators***	***Crew in base***
Kitzbühel Christophorus 4	Austria	OEAMTC	10000	Yes	1070	855	1	yes
Raron	Switzerland	Air Zermatt	1450	Yes	1019	650	1	yes
Zermatt	Switzerland	Air Zermatt	750	Yes	1162	650	1	yes
Sion	Switzerland	Air Glacier	2000	Yes	1932	1200	1	yes
Flycom	Slovenia	FLYCOM	20270	Yes	13	a	1	yes
HEMSSLO	Slovenia	Police	20270	No	274	250	1	close
Aragon	Spain	Police	47000	No	105	275	2	no
MRSLO	Slovenia	Army	20270	No	92	80	3-4	close
PILOTSLO	Slovenia	Army	20270	No	454	165	2	close
Totals					**6121**			

a : HEMS base was closed before 1 year of operation, HEMSLO: actual Slovenian HEMS, MRSLO: Mountain rescue service of Slovenia, PILOTSLO: state pilot project of HEMS.

Time was measured in minutes. The general condition of the patient was defined using the NACA score. This is the most widely used severity score in HEMS in Alpine countries. We also collected data about most important diagnoses like poly-trauma, serious head injury and acute coronary syndrome. However, as a factor with potential impact on the measured times we use the NACA score as a practical and validated measure of the severity of the threats to the patient and the need for emergency on-site medical treatment and transport [23].

Collecting data on the functioning of EMS and other emergency systems is fraught with problems including diversity and missing data, as well as coping with different methods for collection [24]. The data from our HEMS bases was edited and standardized by first author (IT) and then processed with SPSS 16.0 (SPSS Inc, Chicago, IL, USA). Where appropriate mean values and standard deviations were calculated. The statistical analysis used methods of descriptive statistics, univariate and multivariate analyses. To analyze the impact of different factors on activation and approach times, we used nonparametric tests*.* For non-Gaussian data, we used the Kruskal Wallis and Man Whitney tests. The Spearman test was used to measure relations between numeric variables. Variables with statistic significance > 0.05 were included in the multivariate analysis (classification regression method). For classification we used regression tree (CRT). Tree methods are particularly well suited for data mining tasks, where there is usually little prior knowledge of which variables are related and how. Tree methods can often reveal simple relationships between just a few variables that could have easily gone unnoticed using other analytic techniques. We are convinced that no single variable, but the combination of many variables, is important. One of the key properties of the constructed decision tree algorithms is that it is possible to compute relative decisive power or importance of each variable. Importance of each variable is computed over all primary and surrogate splits of this variable in the tree. The Measure of Importance M(X) of an independent variable X in relation to the final tree T, is defined as the (weighted) sum across all nodes in the tree of the improvements that X has when it is used as a primary or surrogate splitter. The independent variable's weights at each split are based on whether the independent variable was the primary splitter (the independent variable on which the parent node was split, where the weight is 1) or a surrogate (where the weight depends on the independent variables ranking as a surrogate. The "Normalized Importance" for a variable is defined as: Normalised M(X) = 100 * M(X) / Maximum Importance. Thus the most important predictor has a normalised importance of 100 [25].

No patient-identifiable data was collected. The study was approved by Medical Ethics Commission of Slovenia and, if necessary, local committees in accordance with national regulations.

## Results

Nine HEMS bases from four mountainous countries took part in the study generating 6121 case reports. Table 1 presents some of the main characteristics of the participating bases.

All HEMS bases operated in a mountainous area, and performed rescue missions both on flat/urban and rural/mountainous terrain. The proportion of missions to specific types of terrain varied between the bases, but the data did not allow the calculation of the ratio between simple (landing the helicopter) and difficult technical rescue procedures, utilizing hovering, hoist or fixed line. 6/9 bases had the capability to perform missions in terrain where landing was not possible by use of a hoist or fixed rope.

Of the 6121 missions analyzed: a doctor was on board and at the site of the incident in all cases. The presence of the doctor, usually an anaesthesist or general practitioner trained in emergency medicine and mountain rescue is a standard in HEMS for all included four countries in the study. In addition, an emergency technician was on board in 44.6%, and a paramedic in 52.2% of cases.

**Figure 1:**
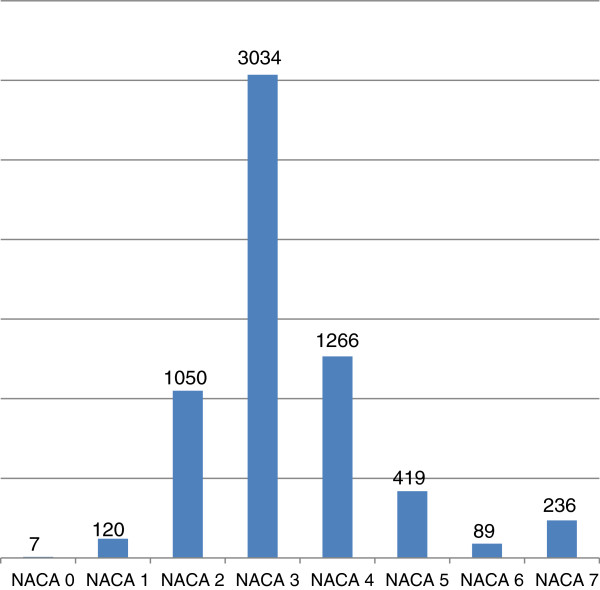
NACA score of included patients.

The average NACA score of victims was 3.3. See Figure 1.

The victim had an injury in 73.0% of cases, serious head injury (Glasgow coma score (GCS) 8 or less) in 7.0%, acute coronary syndrome in 4.2%, and poly-trauma (multiple traumatic injuries, injury severity score (ISS) > 15) in 4.4%. ECG monitoring was undertaken in 36.9%, a 12-channel ECG done in 4.1%, and non-invasive blood pressure and oxygen saturation measured in 40.5% and 42.6% respectively. An intravenous (iv) line was placed in 55.1%, iv analgesia given in 36.1%, oxygen administered in 37.4%, resuscitation because of cardiac arrest of any origin started in 2.8%, intubation in 6,1%, and intubation with rapid sequence induction performed in 2.6% of cases respectively.

Mean activation time (emergency call to HEMS take off) for the participating bases was 7.3 minutes but individual bases varied from 2.9 to 17 minutes. Similarly, mean approach time (emergency call to HEMS arrival at the incident site) was 19.6 minutes with individual bases varying from 10.4 to 45 minutes. See Table 2.

**Table 2:** Activation and approach times of HEMS bases (in minutes)

***HEMS*base**	**Mean activation time**	**SD**	**95% Confidence interval**		**Mean approach time**	**SD**	**95% Confidence interval**	
			**Low**	**High**			**Low**	**High**
Kitzbühel	2.9	1.5	2.9	3.1	10.4	4.4	10.1	10.6
Raron	6.3	5.6	6.0	6.7	18.6	10.4	17.0	19.3
Zermatt	6.4	7.7	6.0	6.9	15.1	10.2	14.5	15.7
Sion	6.9	6.7	6.6	7.2	19.6	10.5	19.2	20.1
Flycom	7.8	2.7	6.2	9.4	29.3	8.9	23.9	34.7
HEMSLO	10.5	5.7	9.8	11.2	31.2	13.0	29.6	32.7
Aragon	16.9	4.3	16.1	17.8	45.0	15.8	41.0	48.1
MRSLO	16.9	12.6	14.2	19.6	41.1	19.9	36.7	45.6
PILOTSLO	17.0	7.2	16.4	17.7	38.5	12.5	37.4	39.7
All	7.3	7.3	7.1	7.5	19.6	13.2	19.3	19.9

State owned helicopter operators (police or army) have significant longer activation (14 minutes versus 4 minutes, p < 0,001) and approach times (36 minutes versus 15 minutes, p < 0,001) in comparison to non state (private) owned operators.

The correlation between activation and approach times is described in Table 3.

**Table 3:** Nonparametric correlation coefficient between approach time, activation time and flight time

		**Activation time**	**Flight time**
Approach time	Spearman rho	0.765^**^	0.867^**^
	p	<0.001	<0.001
	N	6055	6055
Flight time	Spearman rho	0.408^**^	
	p	<0.001	
	N	6055	

Variables activation and approach time are generic dependant, because activation time is part of approach time. The difference is the flight time, the time the helicopter takes to fly from the base to the incident site. The correlation coefficient between activation and approach time is 0.77, so longer approach times are not always the result of longer activation times. The correlation between approach and flight time is higher (0.87), so the duration of approach time is more dependant on flight time.

Some features of HEMS bases have a statistically significant impact on the duration of both the approach and activation time. Statistically significant (p < 0.001) short activation and approach times are in HEMS bases, where:

Helicopter operator is not state owned (not police or army)

HEMS is integrated in state or region EMS system

All HEMS crew members are together at the same location at the base (The distance between helicopter and crew was 20 – 100 meters)

Doctors come from state or private health institutions

HEMS performing organization is private owned

Helicopters are dedicated only for HEMS

Operation area is around 10.000 km^2^

HEMS team is activated by an independent dispatcher who is also in charge of making ‘take-off’ decisions

Dispatching is performed from special center of regional government

There is only one mediator (the dispatcher) in the emergency call

Helicopter is equipped with hoist and/or fixed line (short haul)

HEMS organization has more than one HEMS bases

HEMS base has only one HEMS team

There are spare helicopters at the base

Closest neighboring HEMS base is around 90 km away

HEMS organization is about 20 years old

Average number of missions per year is more than 650

Modern helicopters with short ignition time and faster cruising speed are used (In our study Eurocopter EC 135)

Activation time depends more on above mentioned variables than approach time because, as we have seen, activation time is part of the approach time and is more likely to be the result of organizational, technical or some other factor than approach time which is more related to helicopter velocity and distance between base and the incident site. In our study we didn’t have the data about distances between HEMS base and incident site and the average velocity of the helicopter flight for every mission.

The condition of the patient defined by NACA score has no impact on activation and approach times.

**Figure 2:**
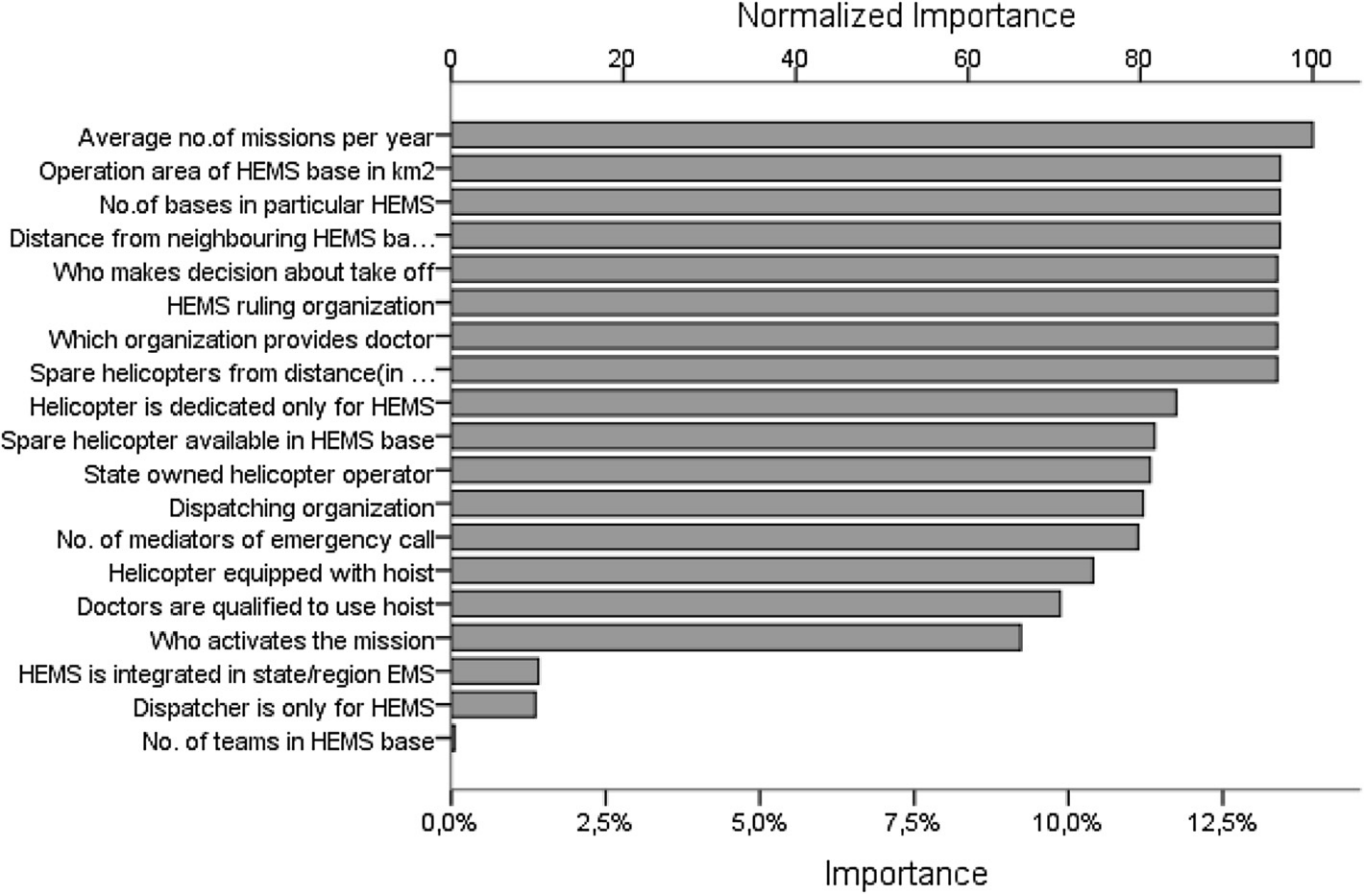
The rank of independent predictors by importance to the activation time (both axes represent arbitrary units of importance and normalized importance, see Methods).

With CRT we identified which variables have the strongest influence on activation times (Figure 2).

**Figure 3:**
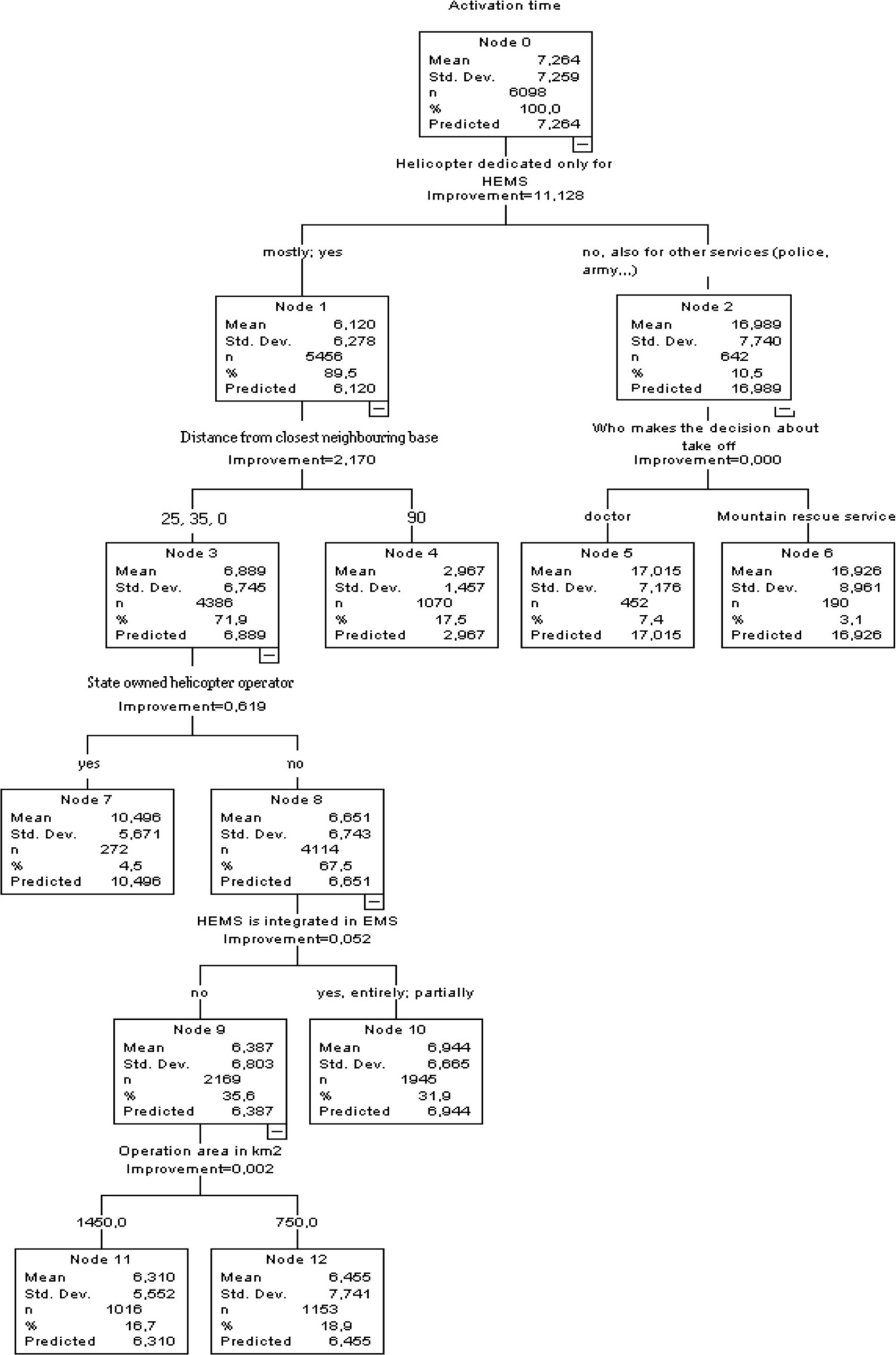
Regression tree classification with the display of activation time improvements.

Improvements of activation time are shown on Figure 3.

The classification shows improvements of activation time on the basis of choosing only such categories of variables, which have as much traces in other categories of variables. This process has two goals:

1) Selection of subgroups or categories of variables, which have shortest activation times. The process of this selection of variables is terminated when further differentiation shows only non significant differences in activation time.

2) Selection of those categories of variables, into which we can pack as many cases of our sample as possible. This is the way in which we increase empiric validity of classification.

The classification regression model of activation time, shows that the first step –the variable with most on shortening of activation time-, is when helicopter is dedicated only to HEMS. This shortens activation time from 17 minutes to 6.1 minute. Following the right branch, the next step is the variable describing the distance of neighboring bases in km. The base with longer distance from a neighboring base (90 km), has shortest activation time – shorter than 3 minutes, which is less than bases with shorter distance from neighboring base, where average activation time is 7 minutes, but there are more sample cases in this cluster. The explanation of second step of differentiation, with an average gain of activation shortening of a little more than 2 minutes, most probably involves geographic/regional characteristics of the HEMS bases. Cluster with average activation time less than 3 minutes is specific and is not continued in next steps of differentiation. The third step, the variable “is HEMS state operated or not”, gains 4 minutes if HEMS is not state operated. If we continue on the branch of cases, where HEMS is not state operated, we can additionally shorten the activation time with the variable “is HEMS integrated or not in EMS of the region”. Activation time is shorter by an average of more than half minute if it is not integrated in EMS. Lastly, the fifth step, where we exhaust the resources to possibly shorten the activation time, the variable concerning the size of operating area of HEMS base gives a shorter activation time the HEMS base has a greater operating area (1.450 km2) compared with bases with smaller (750 km2) operating area.

## Discussion

This is the first international study on activation and approach times of HEMS and the factors that influence these times. We found large differences in activation and approach times between the nine HEMS bases in four alpine countries studied. Among 32 factors we found 17 factors impacted on the activation and approach times. Though not all national HEMS providers (except in Slovenia) were included, the results of our study supported many of recommendations from ICAR MEDCOM [16,26].

From the patient’s point of view the care of critical illness or injury should be a well coordinated continuum which begins with their interaction with emergency call dispatchers and the response of the (H)EMS team [3]. Most of the calls to Alpine HEMS bases are treated as emergency calls despite many, even most, of the cases being non-time critical from medical point of view [7,16,26]. Fast activation of HEMS team is a necessary doctrine as the majority of primary interventions in mountainous areas are regarded as emergency, if not from medical, then from other (environmental, etc.) points of view. Another reason is that before the mission in many, or even most cases the data from the accident site is scarce or unreliable or both. Finally, faster activation and approach times translates into a faster return to the base to be ready for a new mission. In support of these reasons we didn’t find any association between NACA score and activation or approach times.

Different organization solutions mean different performance even with the same resources (crew, helicopters, medical equipment, etc.) and the same locations. We found an almost six fold differences between shortest (2.9 minutes, Kitzbuehel) and longest (17.0 minutes, PILOTSLO) mean activation time. Similar differences were found in mean approach times (shortest-Kitzbuehel 10.4 minutes, longest-Aragon 45.0 minutes). Some international and regional standards or recommendations suggest a maximum activation time of 5 minutes in ideal conditions [1,16,22,26,27]. Only Kitzbuehel base reached this standard though all three Swiss bases came close at activation times of under 7 minutes. There are many factors in addition of factors included in our study, which may impact activation time on each mission (inadequate information from the scene, communication problems, the necessity of longer safety checks etc.) but we believe that an average activation time shows the ability of HEMS team to respond as fast as possible to an emergency situation.

Standards and recommendations also suggest an approach time of less than 20 or 25 minutes even in mountainous areas as an ideal goal from a medical perspective [1,16,22,26,27]. Such average approach times are only possible for HEMS bases covering a range of ≤ 50 km in diameter. It is well known that such maximum operating area for a mountain rescue helicopter is unrealistic in most countries except some European countries. The minimal time standard should be 'as fast as feasible without compromising safety' [1,16,26]. As HEMS missions in the mountains are especially challenging and place unique demands on the persons, organizations and resources involved [7] safety must be the highest priority in mountain rescue [16,28]. We strongly emphasize the primary importance of safety over time and even the medical condition of the patient.

Privatization of (H)EMS is a controversial topic [29]. In our research, HEMS bases with private owned helicopter operators had significant shorter activation and approach times in comparison to state owned operators (police and army). Similar results have been found in Finland and Czech republic [30]. Activation of state owned helicopters, especially of army helicopters is more complicated and time consuming mostly because of bureaucratic reasons. Another reason for their longer times is that they are usually not used solely for HEMS, so they can be busy with other duties and not always immediately available for HEMS missions. Similarly, we found that HEMS bases using helicopters that are used only for HEMS have significantly shorter times reinforcing that HEMS helicopters should be used only for HEMS [16].

We found shorter times when HEMS is integrated in EMS of particular region or state. This integration results in better dispatching, more effective cooperation of services and persons, and greater efficiency from using all available resources. This has been widely described in the literature previously [9,19,26,27,31-35]. The dispatching system has an important influence on effectiveness of (H)EMS including activation times [3,31,33,36,37]. In our study, dispatching by regional (civil) organizations resulted in significantly shorter activation times in comparison with army or police dispatch centers. Activation times are also strongly influenced if a dispatcher can activate the HEMS team; if other organizations (mountain rescue service, police or army) are involved in dispatching and the decision making process, activation times are longer. Most HEMS helicopters operating in Europe are activated through central dispatching centers, where all emergency services are coordinated and this seems to be an optimal solution [38].

Confirming our expectation, we found that the number of intermediators involved in the emergency call has strong influence on activation times. HEMS bases with only one intermediator had shortest activation times; more intermediators cause delays in activation time [31]. For a single emergency call, modern dispatch systems should need only one dispatcher, who performs triage and organizes the emergency intervention of all the services required for completion of the mission [31,33,39]. For this system to work criteria for rational use of helicopters in EMS are necessary [36,38]. Dispatch and activation criteria for pre-hospital critical care services are a priority for future research [37].

The location of the HEMS team members influenced the length of activation time. HEMS bases where all team members were located together close to the helicopter had the shortest activation and approach times. The distance between team members and helicopter within the HEMS base could be important, but in our study the absolute distance waried between 20 and 100 metres and would have had a minimal effect on activation time. Team members in different locations, even in the same HEMS base, increased the pre-take off phase, as did the distance from the helicopter. A ‘Rendezvous’ system (team members in different locations away from the HEMS base) is not recommended from a safety and timing perspective [16,31].

Two simultaneously working teams in one HEMS base did not result in a shorter activation time perhaps reflecting the rarity of simultaneous missions, where it would be expected that two teams would be the advantage. Also missions could have been diverted to neighboring HEMS bases.

The availability of a spare helicopter in the same HEMS base (one variable) or in the neighboring HEMS base (another variable) had an impact on mean activation and approach times, despite the rarity of malfunctions of modern, well-maintained helicopters.

The distance from neighbor HEMS base was found to have substantial impact on observed times. A base with a neighbor 90 km apart had the shortest times. We didn’t find an explanation for this.

Larger operators running more than one HEMS base have shorter times perhaps reflecting better organization and coordination, and more experience.

The size operational area of the HEMS base is second most influential factor for shortening the both activation and approach times. Bases with largest operation areas (20.000 and 47.000 km^2^) have longest times. Two Swiss bases covering areas between 750 and 2000 km^2^ have shorter times, but shortest times are in Kitzbuehel covering an area of 10.000 km^2^. An explanation for the short times in some of the bigger areas includes the effect of more annual cases as a result of a bigger area. Existing literature [31] recommends the operating area should be around a radius of 50 km (around 8.000 km^2^), with a maximum of 70 km (around 15.000 km^2^). Optimization of number and distribution of HEMS bases in a particular region or state is a demanding process, especially in times of economy crisis. Cost benefit compromise is necessary in most countries [12,34].

Average number of missions per base per year was found to be the most influential factor for short activation times. We think responding as fast and as rationally as possible has developed in response to the greater demand resulting in greater effectiveness.

Crew configuration is controversial topic in HEMS. The role of doctors in (H)EMS is well recognized by many authors [27,34,40-42] but is still discussed by others. The doctor is expected to provide a higher level of diagnostic/treatment procedures and more actively participate in organizing and coordinating a mission [16]. In our study doctors were present in all missions; we did not enquiry into the specialization of the doctors though most appear to be anesthetists or general practitioners trained in emergency and mountain rescue [43]. Activation and approach times are influenced by the source of the doctors. The longest times were when a Mountain Rescue Service (MRS) provides doctors; the shortest when doctors come from private or state health institutions. We think that voluntary organizations, such as MRS, may be less efficient organizations and use a rendezvous system more often.

HEMS bases where helicopters are equipped with a hoist, or had fixed line (short haul) capability, and the ability of doctors and other rescue personnel to use it, had shorter activation and approach times. In Alpine regions these results are to be expected, as their use will speed up deployment the HEMS team to the site of the accident and we recommend their inclusion in standards for HEMS operating in the mountains to minimize the delays in getting a doctor to the victim [31,42-46]. Inadequate equipment for the specific mission could prolong approach time, but in our bases helicopters have standardized rescue and medical equipment that cover all needs according to ICAR MEDCOM standards [16], with the exception that in three bases a hoist/fix line was not available.

As expected, HEMS bases using modern helicopters (in our sample only Eurocopter EC 135) with their short pre take off phase have shorter activation times then bases using only older types of helicopters. Helicopter production and sale is only partially influenced by EMS demands [47].

Exclusively stable financing of HEMS is connected with longer activation times. Market principles including competition perhaps encourage HEMS providers to reach higher performance standards [48,49].

### Limitations

The participating HEMS bases were self-selected and may not represent other non-participating bases and services. In Austria, Switzerland and Spain not all of the national HEMS providers are included. However, the study does show the feasibility of achieving short activation and approach times.

Bias from a retrospective, self-reported questionnaire study cannot be excluded.

Data to calculate the ratio between simple retrieval of the victim where the helicopter can land at the incident site and those requiring a difficult retrieval (hovering and hoist, fixed line and technical rescue procedures) was not possible. Differences in this ratio between bases would be expected to have some influence on the approach times.

There are other factors within each base that may have an impact on the time intervals and were not accounted for in our research. These include the distance of the crew from the helicopter, the number of the crew, the actual flight distance and the altitude flown on each mission etc.

## Conclusions

For the severely injured or ill patient, survival in rural and especially in environmental challenging conditions is often time dependent and helicopters can significantly shorten rescue missions. There is a great variation in HEMS activation and approach times suggesting that some HEMS can improve. In our sample of 6121 rescue missions from nine HEMS bases in four mountainous countries we found 17 factors associated with activation and approach times. Other factors which may have an impact on the time intervals and were not accounted in our research, should be investigated in future research. The results of our study supported many of recommendations made by ICAR MEDCOM. We suggest that HEMS organization operating in the mountains should operate within these recommendations and contribute to further work of the ICAR Air Rescue Commission and the Commission for Mountain Emergency Medicine.

Further research is urgently needed to compare HEMS with terrestrial EMS and mountain rescue services, particular in understanding the effect of technical terrain and to validate dispatch criteria Medical treatment and outcome quality indicators should be collected and the factors that influence them researched. Uniform collecting of data in (H)EMS of different countries with as many as possible factors which may have an impact on quality of (H)EMS, would facilitate similar multi-centre research projects including comparison between countries.

In general, efficient HEMS is a combination of expensive highly technical material (helicopters, communication etc.) and, even more importantly, a well-organized , trained and educated human resource (helicopter crew, dispatch centers crew etc.). Safety is of primary importance; even more important than time and the condition of the patient.

## Abbreviations

CRT: Classification and regression tree; HEMS: Helicopter emergency medical service; HEMSLO: Actual HEMS base in Slovenia, operating mostly outside mountains with police air force as a helicopter operator; ICAR: International Commission for Alpine Rescue; ICAR MEDCOM: ICAR Commission for Mountain Emergency Medicine; ISS: Injury severity score; MRSLO: Mountain rescue service of Slovenia; OEAMTC: Austrian Automobile, Motorcycle and Touring Club; PILOTSLO: Pilot project, a predecessor of HEMSLO, with army air force as a helicopter operator.

## Competing interests

The authors declare that they have no competing interests.

## Authors’ contributions

IT conceived, organized and performed most of the study including writing the manuscript. MV participated in the design of the study and performed most of the statistical analysis. JE participated in writing the manuscript including language check. OR and GS provided most of the data. JK was the supervisor of the study and contributed to the writing of the study. All authors read and approved the final manuscript.

## Authors’ information

IT is leading author of ICAR MEDCOM Medical Standards for Mountain Rescue Operations using Helicopters, JE and OR are coauthors of these standards. IT, OR, GS and JE are very experienced mountain rescue doctors, performing both HEMS and terrestrial rescue. They also participated in writing some more ICAR MEDCOM recommendations and other mountain emergency medicine related articles in SCI magazines.
